# Enhanced Dielectric Performance of P(VDF-HFP) Composites with Satellite–Core-Structured Fe_2_O_3_@BaTiO_3_ Nanofillers

**DOI:** 10.3390/polym11101541

**Published:** 2019-09-21

**Authors:** Yongchang Jiang, Zhao Zhang, Zheng Zhou, Hui Yang, Qilong Zhang

**Affiliations:** School of Materials Science and Engineering, State Key Lab Silicon Mat, Zhejiang University, Hangzhou 310027, China; fengbaiwutong@163.com (Y.J.); 21626027@zju.edu.cn (Z.Z.); zhengzifd@126.com (Z.Z.); yanghui@zju.edu.cn (H.Y.)

**Keywords:** nanoparticles, polymer composites, interfacial polarization, dielectric properties

## Abstract

Polymer dielectric materials are extensively used in electronic devices. To enhance the dielectric constant, ceramic fillers with high dielectric constant have been widely introduced into polymer matrices. However, to obtain high permittivity, a large added amount (>50 vol%) is usually needed. With the aim of improving dielectric properties with low filler content, satellite–core-structured Fe_2_O_3_@BaTiO_3_ (Fe_2_O_3_@BT) nanoparticles were fabricated as fillers for a poly(vinylidene fluoride-co-hexafluoropropylene) (P(VDF-HFP)) matrix. The interfacial polarization effect is increased by Fe_2_O_3_ nanoparticles, and thus, composite permittivity is enhanced. Besides, the satellite–core structure prevents Fe_2_O_3_ particles from directly contacting each other, so that the dielectric loss remains relatively low. Typically, with 20 vol% Fe_2_O_3_@BT nanoparticle fillers, the permittivity of the composite is 31.7 (1 kHz), nearly 1.8 and 3.0 times that of 20 vol% BT composites and pure polymers, respectively. Nanocomposites also achieve high breakdown strength (>150 KV/mm) and low loss tangent (~0.05). Moreover, the composites exhibited excellent flexibility and maintained good dielectric properties after bending. These results demonstrate that composite films possess broad application prospects in flexible electronics.

## 1. Introduction

Polymer dielectric materials are extensively applied in flexible electronics and energy storage devices owing to their merits of outstanding flexibility, ease of processing, light weight, and low cost [1,2,3,4]. Despite these merits, the dielectric permittivity (*ε_r_*) of most polymers is quite low (<10). Two main strategies have been developed by researchers to enhance the dielectric permittivity [5,6,7,8,9,10,11,12]. One is incorporating ceramic fillers with intrinsically high dielectric constants (e.g., BaTiO_3_, Ba_x_Sr_1-x_TiO_3_, CaCu_3_Ti_4_O_12_) [13,14,15,16,17,18,19,20] into the polymer matrix; the other strategy is employing conductive fillers, including metals (e.g., Ag, Ni, Al) [21,22,23,24,25], carbon materials (e.g., carbon nanotubes, graphene) [26,27,28,29,30,31,32], semiconductors (e.g., ZnO) [33], and conductive polymers (e.g., polyaniline (PANI)) [34,35,36,37]. With ceramic/polymer composites, the merits of high *ε_r_* from ceramic fillers and high breakdown strength from polymers are combined. However, ceramic fillers with a large amount of additions (>50 vol%) are usually needed to obtain a high *ε_r_*, which can seriously affect flexibility and mechanical properties. Therefore, it is worth studying this problem to further improve the dielectric properties of composites with low filler content.

One type of n-type semiconductor is α-Fe_2_O_3_ (band gap: 2.1 eV). It has been studied extensively in pigments, lithium-ion batteries, gas sensors, and photoelectrochemical water splitting [38,39,40]. It has also attracted the attention of researchers for optimizing the dielectric performance of pure polymers by utilizing α-Fe_2_O_3_. Thakur et al. [41] reported in-situ-synthesized Fe_2_O_3_ in poly(vinylidene fluoride) (PVDF), which promotes the formation of β-PVDF and enhances *ε_r_* through interfacial polarization. Hayashida [42] incorporated α-Fe_2_O_3_ into ten kinds of polymer matrices and studied its influences on the dielectric properties at 40–160 °C. The research showed that the *ε_r_* of composites could be raised due to interfacial polarization induced by free electrons in α-Fe_2_O_3_ particles. In addition, constructing satellite–core-structured fillers for the polymer matrix was considered to be an effective approach for enhancing the dielectric performance. This structure combines two kinds of fillers by loading one filler onto the surface of another. For example, Ag@BT fillers [43] and SnO_2_@BT [44] fillers were fabricated by former researchers and enhanced dielectric properties were obtained compared with pristine BT fillers.

In this work, with the aim of improving dielectric properties with low filler content, Fe_2_O_3_@BT nanoparticles were fabricated as fillers to prepare Fe_2_O_3_@BT/P(VDF-HFP) and (FB/P(VDF-HFP)) composites. Satellite–core-structured Fe_2_O_3_@BT introduces extra interfaces, so the interfacial polarization and *ε_r_* of composites are enhanced. Besides, the satellite–core structure of Fe_2_O_3_@BT prevents the direct contact of Fe_2_O_3_ particles with each other in the polymer matrix, so the loss tangent remains relatively low. 

## 2. Materials and Methods

### 2.1. Materials

N, N-dimethylformamide (DMF) and Barium titanate (BaTiO_3_, BT) were bought from Aladdin (Shanghai, China). P(VDF-HFP) and Ferric nitrate nonahydrate were supplied by Sinopharm (Shanghai, China) and Sigma-Aldrich ( Shanghai, China), respectively.

### 2.2. Synthesis of Satellite-Core-Structured Fe_2_O_3_@BT Nanoparticles

The 0.303 g ferric nitrate nonahydrate was first dissolved in deionized water (100 mL). Then, 0.700 g BT nanoparticles were dispersed into this solution via sonicating and stirring. The molar ratio of BT/Fe was 4:1. The solution was stirred at 75 °C for 5 h, and cleaned with deionized water. After drying under vacuum, FeOOH@BT nanoparticles were obtained. The generated powder was heated at 550 °C for 2 h in air. Satellite–core-structured Fe_2_O_3_@BT nanoparticles were then generated.

### 2.3. Fabrication of Fe_2_O_3_@BT/P(VDF-HFP) Composites

A stoichiometric amount of Fe_2_O_3_@BT nanoparticles were distributed into dimethylformamide (DMF) via stirring and ultrasound. P(VDF-HFP) was then added and vigorously stirred for 12 h. The feeding ratio of P(VDF-HFP)/DMF was 1 g:15 mL. The composite films were then prepared through drop casting onto clean glass plates. The composites were kept at 60 °C to eliminate DMF, and then heated to 200 °C (5 min) and quenched in ice water. BT/P(VDF-HFP) and pure polymer were also generated.

### 2.4. Characterization

Scanning electron microscopy (SEM) (SU-8010, Hitachi, Japan) and transmission electron microscopy (TEM) using a Tecnai G2 F20 (FEI, Hillsboro, OR, USA) (accelerating voltage: 200 kV) with energy dispersive spectroscopy (EDS) were applied to examine the morphology of composites and particles. The elemental composition of nanoparticles was observed using X-ray photoelectron spectroscopy (XPS) with an Escalab 250Xi. XRD (X’ Pert PRO, PANalytical, Netherlands) using Cu Kα radiation was performed to identify the components of particles and composites. Differential scanning calorimetry (DSC) was tested by TA-Q200 at 90–190 °C (10 °C/min, nitrogen atmosphere). Dielectric performances were measured with an 4294 impedance analyzer (Agilent, Palo Alto, CA, USA) from 10^2^–10^6^ Hz (silver electrode, diameter: 4 mm, thickness: 100 nm). A dielectric strength tester (CS2674AX, Nanjing Changsheng, Nanjing, China) was employed to test the Direct Current (DC) breakdown strength under a direct current voltage ramp of 200 V s^−1^ at 25 °C.

## 3. Results and Discussion

### 3.1. Morphology and Structure of Fe_2_O_3_@BT Nanoparticles

**Figure 1:**
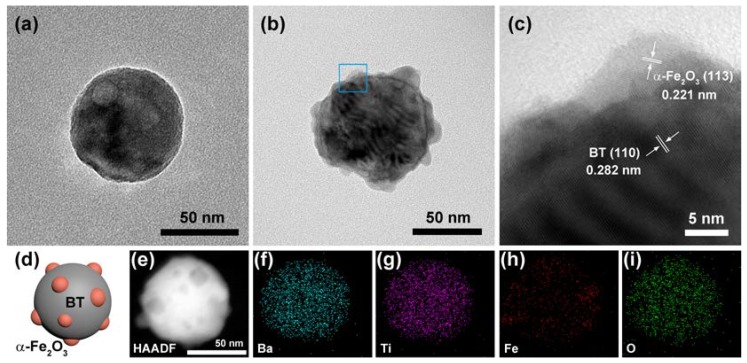
(**a**) Transmission electron microscopy (TEM) photo of a BT nanoparticle. (**b**) TEM and (**c**) HRTEM photos of a Fe_2_O_3_@BT nanoparticle. (**c**) Partially enlarged image of the blue square area in image (**b**). (**d**) Schematic illustration of a Fe_2_O_3_@BT nanoparticle. (**e**–**i**) HAADF-STEM image with mapping images of a Fe_2_O_3_@BT nanoparticle. The scale of the images (**f**–**i**) is the same with that of image (**e**).

Figure 1 presents the TEM photos of Fe_2_O_3_@BT nanoparticles, as well as the EDS elemental mapping photos. The pure BT nanoparticles are spherical, with a diameter of about 50–100 nm. Fe_2_O_3_ nanoparticles (5–10 nm) decorated on BT and the satellite–core-structured Fe_2_O_3_@BT nanoparticles are formed. As shown in the High Resolution Transmission Electron Microscope (HRTEM) image, the lattice fringe areas with 0.221 nm and 0.282 nm spacing are assigned to (113) and (110) planes of α-Fe_2_O_3_ and BT (JCPDS 75-0462, 33-0664), respectively [45,46]. The structure of Fe_2_O_3_@BT nanoparticles is illustrated in Figure 1d. EDS results further reveal the distribution of Fe_2_O_3_. It is shown that Ba, Ti, and O are homogenously distributed on the surface of nanoparticles. However, the amount of Fe is much less and its distribution is locally concentrated, corresponding to the satellite–core structure.

**Figure 2:**
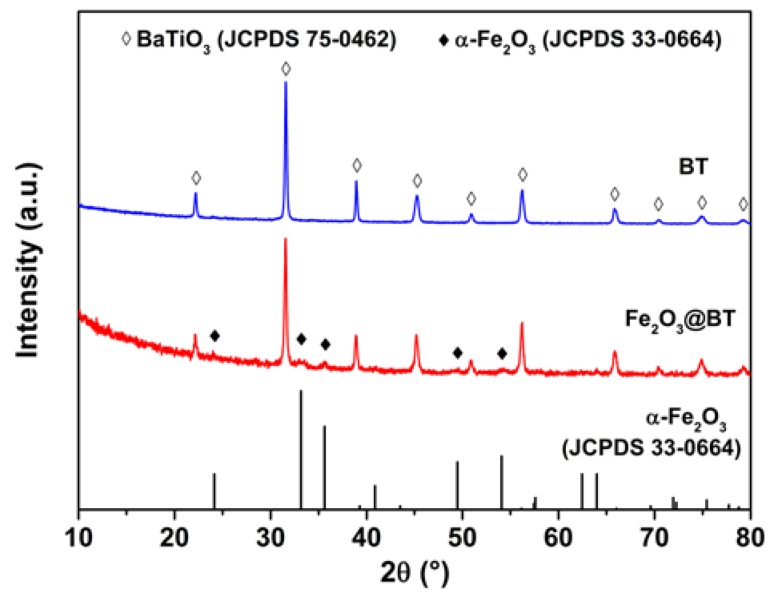
X-ray diffraction (XRD) patterns of Fe_2_O_3_@BT and BT nanoparticles.

Figure 2 presents XRD patterns of BT and hybrid particles. Characteristic peaks of BaTiO_3_ (Joint Committee on Powder Diffraction Standards (JCPDS) 75-0462) are obviously shown in hybrid particles. Moreover, some weak peaks at 24.1°, 33.2°, 35.6°, 49.5°, and 54.1° are also observed, corresponding to the (012), (104), (110), (024), and (116) planes of α-Fe_2_O_3_, respectively. No other phases of Fe_2_O_3_ are shown, which indicates that only α-Fe_2_O_3_ is obtained after calcination at 550 °C [47,48,49].

**Figure 3:**
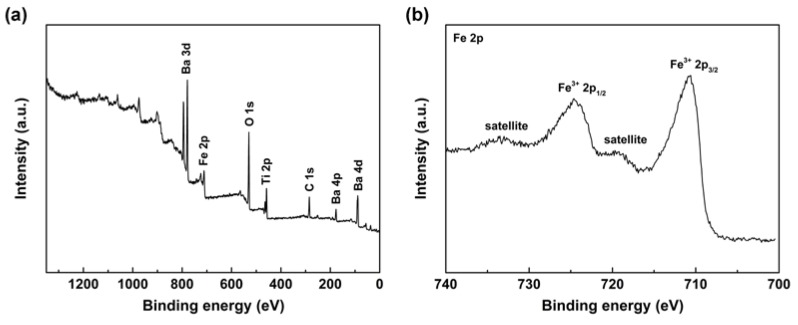
X-ray photoelectron spectroscopy (XPS) spectra of Fe_2_O_3_@BT nanoparticles: (**a**) survey scan, (**b**) Fe 2p.

To further analyze the elemental composition, XPS is conducted on Fe_2_O_3_@BT nanoparticles. As shown in Figure 3a, characteristic peaks of Ba, O, Fe, C, and Ti are shown in survey scan spectra. In Figure 3b, the peak at 724.6 eV and 710.9 eV correspond to Fe^3+^ 2p_1/2_ and Fe^3+^ 2p_3/2_ peaks, together with two satellite peaks at 733.5 eV and 719.2 eV. The binding energy difference between 2p_1/2_ and 2p_3/2_ is 13.7 eV. Besides, characteristic peaks are not observed for Fe^2+^ [50,51,52,53]. These results indicate that the element Fe in nanoparticles exists in the form of Fe^3+^, which means Fe_2_O_3_ is synthesized. In addition, the color of the powders is red-brown, which is consistent with that of Fe_2_O_3_.

### 3.2. Structure and Morphology of Fe_2_O_3_@BT/P(VDF-HFP) Composites

**Figure 4:**
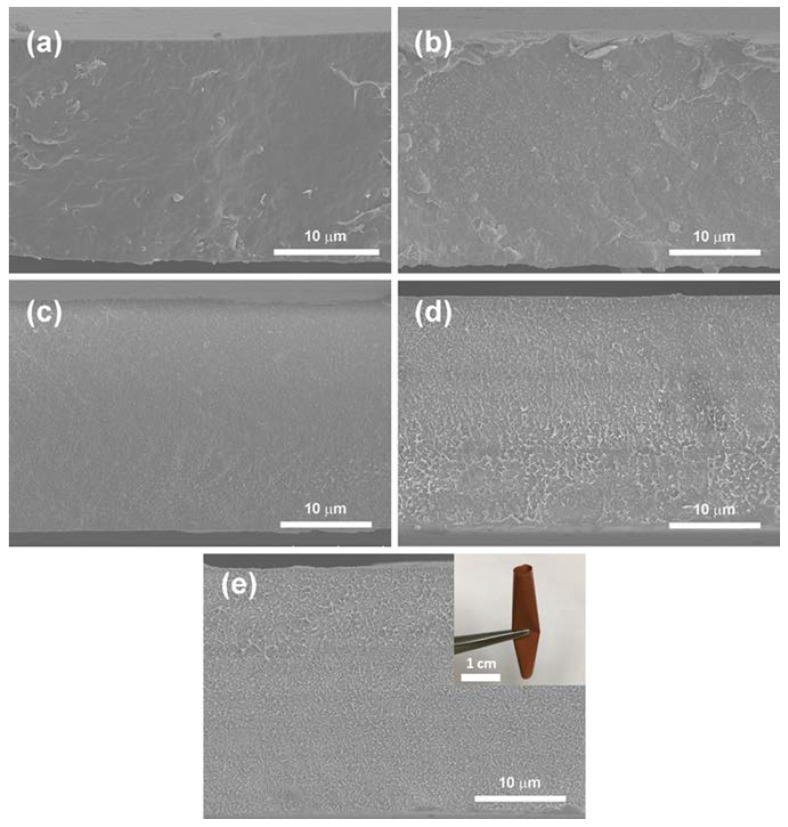
SEM images of cross-sectional film of: (**a**) pristine P(VDF-HFP), (**b**) 5 vol%, (**c**) 10 vol%, (**d**) 15 vol%, and (**e**) 20 vol% composites. Inset is the digital photograph of 20 vol% film.

Figure 4 presents cross-section morphologies of composites. Numerous nanoparticle fillers are shown in the polymer. According to the XRD results of the composites, it can be seen that these nanoparticles are Fe_2_O_3_@BT. The nanoparticles are distributed well in P(VDF-HFP) and no apparent void or pore can be observed. In addition, the inset shows the digital photograph of 20 vol% composites, which can still be easily bent and rolled. 

**Figure 5:**
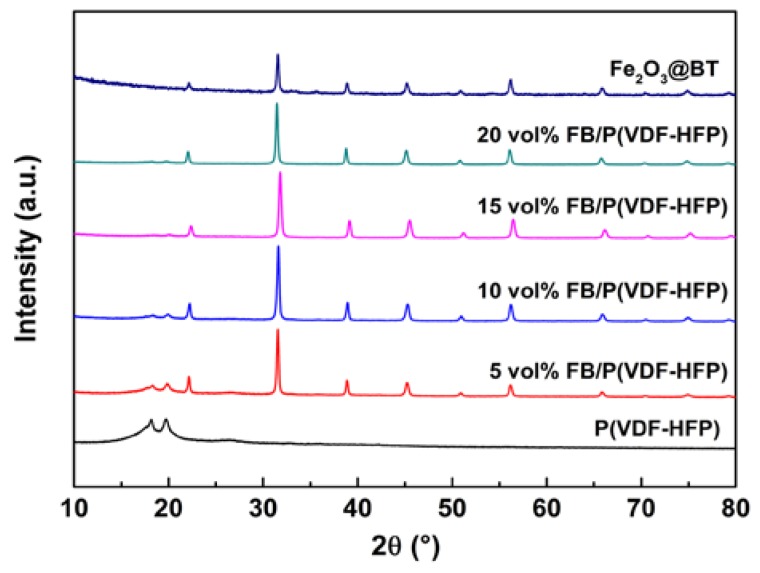
XRD patterns of P(VDF-HFP), Fe_2_O_3_@BT nanoparticles, and FB/P(VDF-HFP) composites.

Figure 5 demonstrates XRD patterns of composites. The three peaks at 18.2°, 19.9°, and 26.5° correspond to the (020), (110), and (021) planes of α-P(VDF-HFP), respectively [54,55]. The hybrid nanofillers peaks can be observed, as well as the matrix peaks. The relative intensity of the matrix peaks decreases as the Fe_2_O_3_@BT increases.

### 3.3. Melting and Crystallization Behavior of Fe_2_O_3_@BT/P(VDF-HFP) Composites

**Figure 6:**
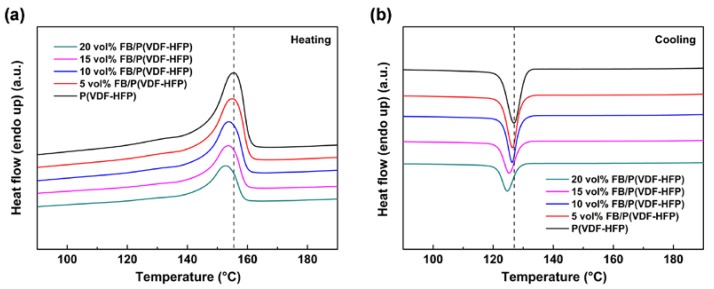
DSC of polymer and FB/P(VDF-HFP) composite (**a**) heating curves and (**b**) cooling curves.

Differential scanning calorimetry (DSC) was performed to analyze the crystallization of the polymer. As is shown in Figure 6a, a melting peak appears in the heating curve for each film, corresponding to the melting process of the polymer. The melting temperature (T_m_) and crystallization temperature (T_c_) decrease as the filler increases. The crystallinity (χ_c_) can be calculated through the formula below:

(1) $$\chi_{c}=\frac{{\Delta H}_{m}}{\left(1-\omega \right)\times {\Delta H}_{m}^{0}}\times 100\%$$

where ΔH_m_ and ${\Delta H}_{m}^{0}$ represent the melting enthalpy of samples and 100% crystallized α-P(VDF-HFP) (93.07 J/g), respectively. Here, ω is the weight fraction of Fe_2_O_3_@BT nanoparticles in composites. 

As shown in Table 1, when the filler content increases, the crystallization peak moves towards lower temperatures and T_c_ decreases gradually. This phenomenon is mainly attributed to the hindering effect of nanoparticle fillers [56,57]. During the crystallization process, the Fe_2_O_3_@BT nanoparticles retard the movement of the polymer chain and impede the progress of crystallization, leading to the decrease of T_c_. Fe_2_O_3_@BT can also act as a heterogeneous nucleation site, facilitating the crystallization. However, the hinderance effect dominates the crystallization process and the influences of heterogeneous nucleation are covered up. When more Fe_2_O_3_@BT nanoparticles are added, the hinderance effect is further enhanced and T_c_ continues to decrease. The final χ_c_ also reduces gradually because of the accumulation of the hinderance effect during crystallization.

### 3.4. Dielectric Properties of Fe_2_O_3_@BT/P(VDF-HFP) Composites

**Figure 7:**
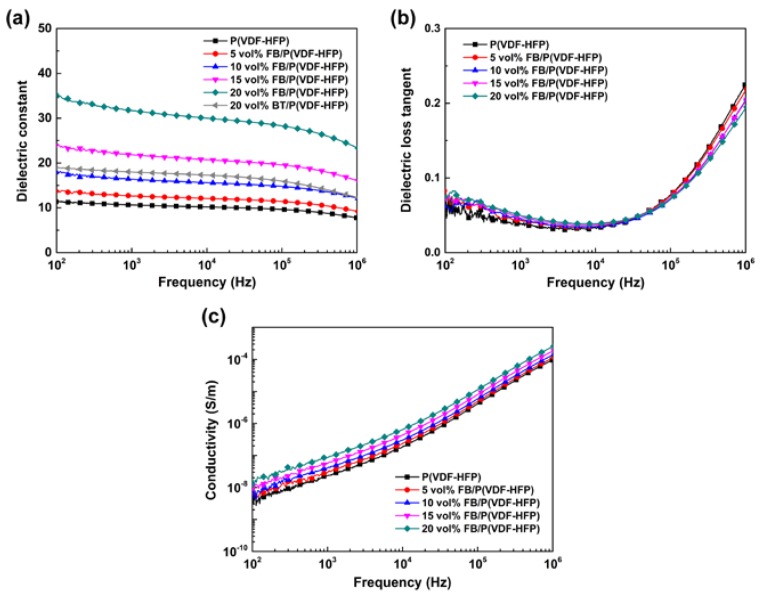
Frequency dependence of (**a**) dielectric constant, (**b**) dielectric loss tangent, and (**c**) conductivity of pristine polymer, BT/P(VDF-HFP), and FB/P(VDF-HFP) composites.

**Figure 8:**
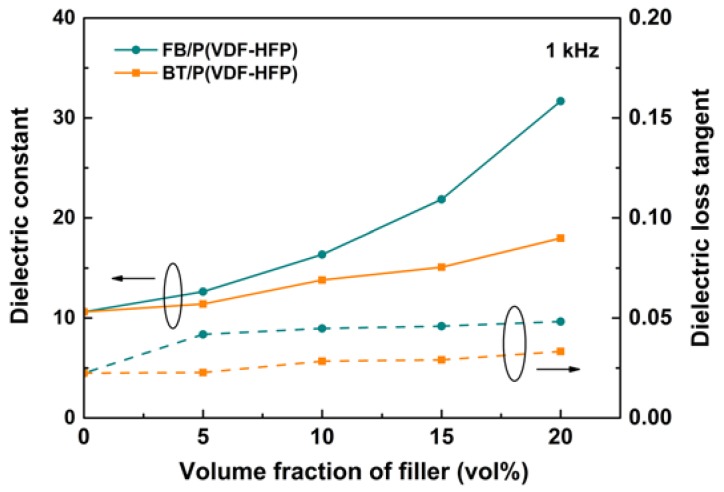
Dielectric properties of the composites filled with Fe_2_O_3_@BT and BT as a function of filler content at 1 kHz.

Figure 7 presents the dielectric characteristics of a pristine polymer, FB/P(VDF-HFP), and 20 vol% BT/P(VDF-HFP). In Figure 7a, the *ε_r_* of each composite decreases as the frequency gets higher. This phenomenon is due to the interfacial polarization relaxation and dipole polarization relaxation at low and high frequencies. To further analyze the influences of Fe_2_O_3_@BT nanoparticles on the dielectric performance of composites, *ε_r_* and tan δ values at 1 kHz of all samples are compared in Figure 8 (left axis). As the content of nanoparticles increases, the *ε_r_* of FB/P(VDF-HFP) is increased notably. The enhancement is larger than in BT/P(VDF-HFP) at the same concentration, which is caused by the interfacial polarization induced by Fe_2_O_3_@BT particles, an important polarization mechanism that occurs in low frequency ranges because of its relatively long time of establishment. When a dielectric is placed in an electric field, the internal free electrons and holes migrate under the electric field and gather at the interfacial area containing two phases, impurities, and defects. Then, dipole moments are generated, and thus, interfacial polarization is induced. In FB/P(VDF-HFP) composites, the satellite–core-structured Fe_2_O_3_@BT nanoparticles introduce extra interfaces, including the Fe_2_O_3_/BT interface, Fe_2_O_3_/P(VDF-HFP) interface, and BT/P(VDF-HFP) interface; semi-conductive Fe_2_O_3_ brings about more charge carriers. Therefore, the interfacial polarization is enhanced by Fe_2_O_3_@BT nanoparticles and the dielectric permittivity of composites is raised. Figure S1 exhibits the dielectric performances of BT/P(VDF-HFP) composites. With 20 vol% nanoparticles added, the *ε_r_* value of Fe_2_O_3_@BT/P(VDF-HFP) is 31.7 at 1 kHz, nearly 1.8 and 3.0 times that of 20 vol% BT/P(VDF-HFP) (18.0) and pure polymer (10.6), respectively. Figure S2 shows that the composite maintains good dielectric performances after bending, which proves the potential application in flexible electronics.

Figure 7b shows the dielectric loss of composites. Tan δ declines at first and then increases for each sample as the frequency gets higher. The increase of tan δ is attributed to dipole polarization relaxation at high frequency. In this range, the establishment of dipole polarization cannot follow the electric field, so the relaxation leads to enhanced loss. The tan δ of composites is lower than the pristine polymer and it continues to decrease when the filler content increases. This phenomenon probably occurs because the Fe_2_O_3_@BT nanoparticles retard the movement of polymer chains, which can decrease the dipole polarization relaxation loss [58,59]. The loss tangent is derived from electric conduction loss and interfacial polarization relaxation at low frequencies. The tan δ values of all samples at 1 kHz are also compared in Figure 8 (right axis). With the increase of nanofiller content, the tan δ is slightly increased, because Fe_2_O_3_ generates many charge carriers. However, the satellite–core structure of Fe_2_O_3_@BT could prevent the direct contact of Fe_2_O_3_ particles with each other in the polymer matrix and suppress the long-range movement of charge carriers; therefore, the tan δ remains low (<0.06). With the addition of 20 vol% Fe_2_O_3_@BT nanoparticles, the tan δ of composites maintains a rather low value of 0.05. The tan δ values of BT/P(VDF-HFP) (20 vol%) and pure polymer are 0.03 and 0.02, respectively (Figure S1). And compared with other BT-based/polymer nanocomposites reported in the previous literature (Table S1), the results of the FB/P(VDF-HFP) nanocomposites reported herein are comparable or better. Figure 7c shows that the conductivity of composites increases when more nanofillers are added. Nevertheless, the conductivity of all composites is lower than 2 × 10^−8^ S/m, proving that the film provides good insulation. 

**Figure 9:**
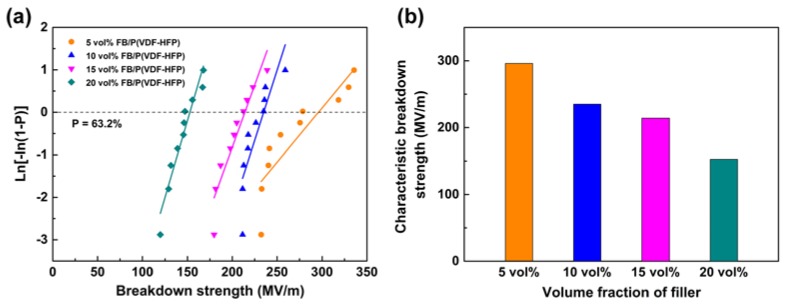
(**a**) Weibull distribution for breakdown strength and (**b**) characteristic breakdown strength of composites.

Breakdown strength (*E_b_*) is also a significant characteristic and determines the energy density and work voltage of composites. Due to the randomness of breakdown events, measured data of *E_b_* is usually further processed by a two-parameter Weibull distribution function [60,61]:

(2) $$P=1-\exp\left[-{\left(\frac{E}{E_{0}}\right)}^{\beta }\right]$$

where P is the cumulative probability of electrical failure, E represents breakdown strength, E_0_ is the characteristic breakdown strength (cumulative failure probability: 0.632), and β is the shape parameter. As shown in Figure 9, breakdown strength decreases as the nanofiller content increases. This phenomenon results from the electrical mismatch between the polymer and the nanoparticles. However, the satellite–core structure of Fe_2_O_3_@BT nanoparticles suppresses the rise of dielectric loss and impedes the formation of conductive paths, so *E_b_* still remains at a relatively high level. The *E_b_* value of the 20 vol% Fe_2_O_3_@BT-filled composite is 152.7 MV/m.

## 4. Conclusions

Satellite–core-structured Fe_2_O_3_@BT nanoparticles were fabricated as fillers to prepare FB/P(VDF-HFP) composites. Fe_2_O_3_@BT nanoparticles show a hinderance effect on the crystallization process of polymers and the crystallization temperature and crystallinity of composite films both decrease as the content of the filler increases. The interfacial polarization effect is enhanced by Fe_2_O_3_ nanoparticles, and thus, the dielectric permittivity of composites is enhanced. The satellite–core structure prevents Fe_2_O_3_ particles from directly contacting each other, so the dielectric loss remains low. With the addition of 20 vol% Fe_2_O_3_@BT nanoparticles, the permittivity value of the composite is 31.7 at 1 kHz, nearly 1.8 and 3.0 times that of the 20 vol% BT and pristine polymer, respectively. Nanocomposites also demonstrate low loss tangent (~0.05) and high breakdown strength (>150 KV/mm). In addition, the composites also exhibit excellent flexibility and maintains good dielectric performances after bending.

## Figures and Tables

**Table 1:** Melting Temperature (T_m_), crystallization temperature (T_c_), and crystallinity (χ_c_) of polymer and FB/P(VDF-HFP) composites.

Sample	P(VDF-HFP)	5 vol% Fe_2_O_3_@BT	10 vol% Fe_2_O_3_@BT	15 vol% Fe_2_O_3_@BT	20 vol% Fe_2_O_3_@BT
T_m_ (°C)	155.5	155.0	153.9	153.7	153.0
T_c_ (°C)	127.0	126.6	126.3	125.4	124.7
χ_c_ (%)	37.3	35.4	33.4	33.2	30.7

## Supplementary Materials

The following are available online at https://www.mdpi.com/2073-4360/11/10/1541/s1. Figure S1: Frequency dependence dielectric performances of BT/P(VDF-HFP) composites. Figure S2: Digital photograph of 20 vol% FB/P(VDF-HFP) composites in (a) unbent and (b) bent statuses. (c) Comparison of dielectric properties of the original 20 vol% FB/P(VDF-HFP) composites and bent composites for 1000 cycles. Table S1: Some research studies related to BaTiO_3_ in PVDF-based composites.
