# Supplementary material for: Enhanced Dielectric Performance of P(VDF-HFP) Composites with Satellite–Core-Structured Fe2O3@BaTiO3 Nanofillers
Source: Polymers (Basel). 2019 Sep 21;11(10):1541. doi: 10.3390/polym11101541 (PMC6835555; doi:10.3390/polym11101541)
Supplement: Supplementary file 1 [file polymers-11-01541-s001.pdf]

Electronic Supplementary Information for:

Enhanced Dielectric Performance of P(VDF-HFP) Composites with Satellite-core Structured Fe_2O_3@$BaTiO_3$ Nanofillers

Yongchang Jiang, Zhao Zhang, Zheng Zhou, Hui Yang, Qilong Zhang*

[1] School of Materials Science and Engineering, State Key Lab Silicon Mat, Zhejiang University, Hangzhou 310027, P. R. China

* Correspondence: mse237@zju.edu.cn

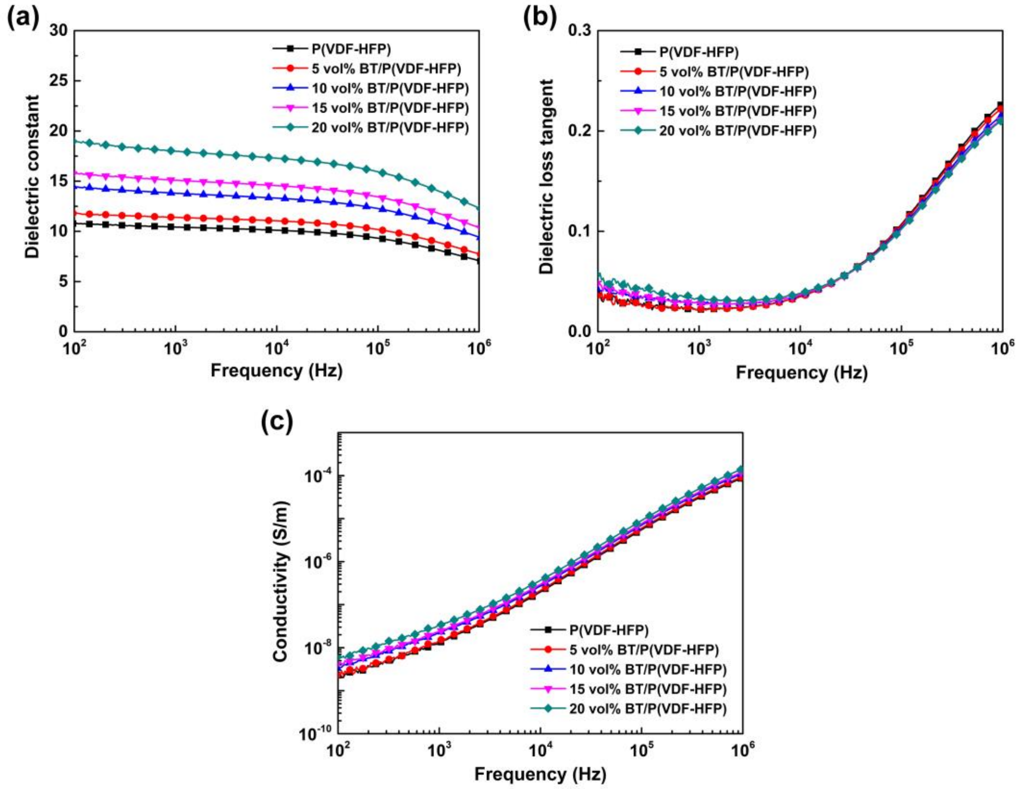

Figure S1. Frequency-dependence dielectric performances of BT/P(VDF-HFP) composites.

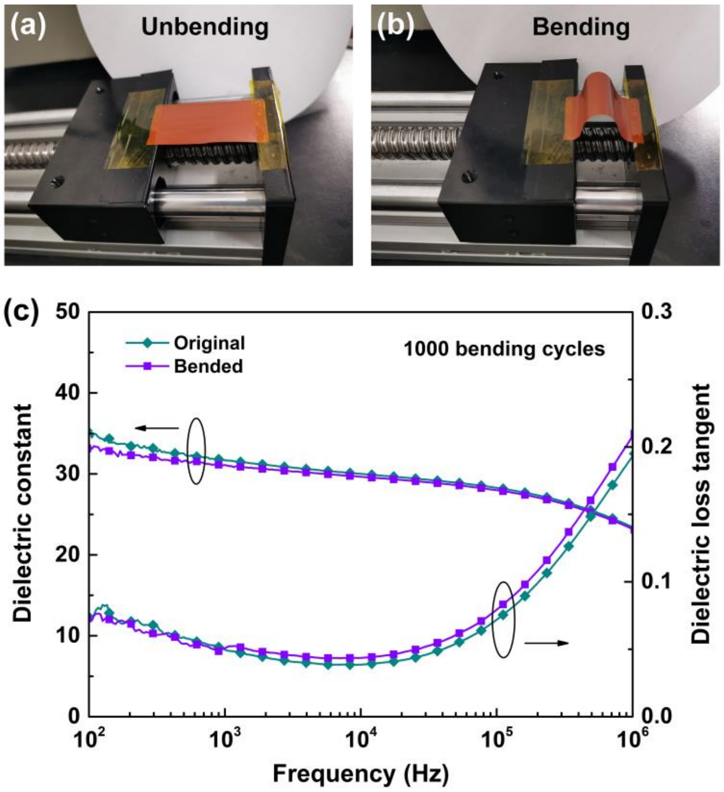

Figure S2. Digital photograph of 20 vol% FB/P(VDF-HFP) composites at (a) unbending and (b) bending status. (c) Comparison of dielectric properties of the original 20 vol% FB/P(VDF-HFP) composites and bended for 1000 cycles.

Table S1. Some researches related to $BaTiO_3$ in PVDF-based composites.

Materials	Filler content	Measuring frequency	Dielectric constant	Dielectric loss tangent	References
Ag@PDA@BT/P(VDF-HFP)	20 vol%	1 kHz	~24	~0.03	[1]
BT@PGMA/PVDF	20 vol%	1 kHz	~18	~0.025	[2]
BT-OH/P(VDF-TrFE)	20 vol%	10 kHz	24	0.036	[3]
Ag@BT/PVDF	56.8 vol%	1 kHz	160	0.11	[4]
SnO_2@BT/PVDF	45 vol%	1 kHz	90	~0.45	[5]
BT-NXT105/PVDF	20 vol%	1 kHz	20.2	~0.025	[6]
BT-PVP/PVDF	55 vol%	1 kHz	77	0.093	[7]
BT@SiO_2/PVDF	2 vol%	1 kHz	12	~0.03	[8]
BT@PANI/P(VDF-HFP)	20 vol%	1 kHz	99.1	0.21	[9]
Ni@PDA@BT NWs/P(VDF-HFP)	20 vol%	1 kHz	48.4	0.23	[10]
Fe_2O_3@BT/P(VDF-HFP)	20 vol%	1 kHz	31.7	0.05	This work

References:

1. Yang, K.; Huang, X.; He, J.; Jiang, P. Strawberry-like core-shell Ag@polydopamine@$BaTiO_3$ hybrid nanoparticles for high-k polymer nanocomposites with high energy density and low dielectric loss. *Adv. Mater. Interfaces* **2015**, *2*, 1500361, doi:10.1002/admi.201500361.
2. Zhu, M.; Huang, X.; Yang, K.; Zhai, X.; Zhang, J.; He, J.; Jiang, P. Energy storage in ferroelectric polymer nanocomposites filled with core-shell structured polymer@$BaTiO_3$ nanoparticles: understanding the role of polymer shells in the interfacial regions. *ACS Appl. Mater. Interfaces* **2014**, *6*, 19644-19654, doi:10.1021/am504428u.
3. Almadhoun, M.N.; Bhansali, U.S.; Alshareef, H.N. Nanocomposites of ferroelectric polymers with surface-hydroxylated $BaTiO_3$ nanoparticles for energy storage applications. *J. Mater. Chem.* **2012**, *22*, 11196-11200, doi:10.1039/c2jm30542a.
4. Luo, S.; Yu, S.; Sun, R.; Wong, C.P. Nano Ag-deposited $BaTiO_3$ hybrid particles as fillers for polymeric dielectric composites: toward high dielectric constant and suppressed loss. *ACS Appl. Mater. Interfaces* **2014**, *6*, 176-182, doi:10.1021/am404556c.
5. Zha, J.-W.; Meng, X.; Wang, D.; Dang, Z.-M.; Li, R.K.Y. Dielectric properties of poly(vinylidene fluoride) nanocomposites filled with surface coated $BaTiO_3$ by SnO_2 nanodots. *Appl. Phys. Lett.* **2014**, *104*, 072906, doi:10.1063/1.4866269.
6. Yu, K.; Wang, H.; Zhou, Y.; Bai, Y.; Niu, Y. Enhanced dielectric properties of $BaTiO_3$/poly(vinylidene fluoride) nanocomposites for energy storage applications. *J. Appl. Phys.* **2013**, *113*, 034105,

doi:10.1063/1.4776740.

7. Yu, K.; Niu, Y.; Zhou, Y.; Bai, Y.; Wang, H.; Randall, C. Nanocomposites of surface-modified $BaTiO_3$ nanoparticles filled ferroelectric polymer with enhanced energy density. *J. Am. Ceram. Soc.* **2013**, *96*, 2519-2524, doi:10.1111/jace.12338.
8. Yu, K.; Niu, Y.; Bai, Y.; Zhou, Y.; Wang, H. Poly(vinylidene fluoride) polymer based nanocomposites with significantly reduced energy loss by filling with core-shell structured $BaTiO_3/SiO_2$ nanoparticles. *Appl. Phys. Lett.* **2013**, *102*, 102903, doi:10.1063/1.4795017.
9. Zhang, Q.; Jiang, Y.; Yu, E.; Yang, H. Significantly enhanced dielectric properties of P(VDF-HFP) composite films filled with core-shell $BaTiO_3$@PANI nanoparticles. *Surf. Coat. Technol.* **2019**, *358*, 293-298, doi:10.1016/j.surfcoat.2018.11.056.
10. Jiang, Y.; Wang, J.; Zhang, Q.; Yang, H.; Shen, D.; Zhou, F. Enhanced dielectric performance of P(VDF-HFP) composites filled with Ni@polydopamine@$BaTiO_3$ nanowires. *Colloids Surf., A* **2019**, *576*, 55-62, doi:10.1016/j.colsurfa.2019.05.039.
